# *Acer tataricum* subsp. *ginnala* Inhibits Skin Photoaging via Regulating MAPK/AP-1, NF-κB, and TGFβ/Smad Signaling in UVB-Irradiated Human Dermal Fibroblasts

**DOI:** 10.3390/molecules26030662

**Published:** 2021-01-27

**Authors:** Yu-Jung Jin, Yura Ji, Young-Pyo Jang, Se-Young Choung

**Affiliations:** 1 Department of Life and Nanopharmaceutical Sciences, Graduate School, Kyung Hee University, Seoul 02447, Korea; yj0831@khu.ac.kr (Y.-J.J.); j5620242@naver.com (Y.J.); ypjang@khu.ac.kr (Y.-P.J.); 2 Department of Oriental Pharmaceutical Science, College of Pharmacy, Kyung Hee University, Seoul 02447, Korea; 3 Department of Preventive Pharmacy and Toxicology, College of Pharmacy, Kyung Hee University, Seoul 02447, Korea

**Keywords:** *Acer tataricum* subsp. *ginnala*, matrix metalloproteinase (MMP), MAPK/AP-1, NF-kB, type I procollagen, TGFβ/Smad

## Abstract

Skin, the organ protecting the human body from external factors, maintains structural and tensile strength by containing many collagen fibrils, particularly type I procollagen. However, oxidative stress by ultraviolet (UV) exposure causes skin photoaging by activating collagen degradation and inhibiting collagen synthesis. *Acer tataricum* subsp. *ginnala* extract (AGE) is a herbal medicine with anti-inflammatory and anti-oxidative effects, but there is no report on the protective effect against skin photoaging. Therefore, we conducted research concentrating on the anti-photoaging effect of *Acer tataricum* subsp. *ginnala* (AG) in UVB (20 mJ/cm^2^)-irradiated human dermal fibroblasts (HDF). Then, various concentrations (7.5, 15, 30 µg/mL) of AGE were treated in HDF for 24 h following UVB irradiation. After we performed AGE treatment, the matrix metalloproteinase1 (MMP1) expression was downregulated, and the type I procollagen level was recovered. Then, we investigated the mitogen-activated protein kinases/activator protein 1 (MAPK/AP-1) and nuclear factor-κB (NF-κB) pathway, which induce collagen breakdown by promoting the MMP1 level and pro-inflammatory cytokines. The results indicated that AGE downregulates the expression of the MAPK/AP-1 pathway, leading to MMP1 reduction. AGE inhibits nuclear translocation of NF-κB and inhibitor of nuclear factor-κB (IκB) degradation. Therefore, it downregulates the expression of MMP1 and pro-inflammatory cytokines such as TNF-α, IL-1β, and IL-6 increased by UVB. Besides, the TGFβ/Smad pathway, which is mainly responsible for the collagen synthesis in the skin, was also analyzed. AGE decreases the expression of Smad7 and increases TGFβRII expression and Smad3 phosphorylation. This means that AGE stimulates the TGFβ/Smad pathway that plays a critical role in promoting collagen synthesis. Thus, this study suggests that AGE can be a functional material with anti-photoaging properties.

## 1. Introduction

Skin tissue, the largest organ in the human body, plays the role of a barrier against harmful external factors: Infectious pathogens, chemical agents, and UV. Therefore, the skin tends to be directly exposed to various diseases and aging. Among harmful agents, UV is the main factor that indisputably damages the skin [1,2]. UV can be divided into three types according to wavelength, UVA (315–400 nm), UVB (280–315 nm), and UVC (100–280 nm). Among them, UVA and UVB can reach human skin by passing through the earth’s atmosphere. UVA penetrates the dermis, and UVB can reach the epidermis and upper side of the dermis. Particularly, UVB is more hazardous biologically than UVA when the skin is exposed to UV at similar irradiation doses. In terms of collagen in extracellular matrix (ECM), UVB upregulates MMPs, the dominant factor degrading collagen fibers. Moreover, it impairs the collagen synthesis pathway through decreasing TGF-β receptor II. Therefore, UVB mainly causes photoaging [3].

UV-induced damage is cumulative and also superimposed on intrinsic aging, leading to old-looking skin. It is called photoaging, characterized by deep wrinkling, dryness, laxity, loss of elasticity, hyperpigmentation, and rough-textured appearance [4]. UV increases hydrogen peroxide and many different free radicals such as superoxide anions, hydroxyl radicals, and singlet oxygen. These are highly reactive and impair biological components such as lipids, proteins, and nucleic acids in cells [5]. Human dermal fibroblasts (HDFs) are the most common skin cells that synthesize collagen fibers that maintain flexibility and physical strength in the dermis. In addition, HDFs interact with collagen fibrils and thus maintain a state of mechanical tension [6]. However, UV irradiation negatively influences the skin by inducing a state of oxidative stress and morphological alteration of HDFs, and regulating the expression of signaling pathways related to skin wrinkles. In the normal state, morphological features of HDFs are flattened and spread. These are in intimate contact with many intact collagen fibrils. On the contrary, in the UV-damaged state, HDFs have a collapsed structure and have difficulty associating directly with fragmented collagen fibrils [7,8].

In the skin, impairment of type I procollagen is dependent on the MMP1 expression, which is a zinc-dependent endopeptidase that degrades type I collagen specifically. It is mainly regulated by the upper signaling cascade, such as MAPKs, extracellular signal-regulated kinase (ERK), c-Jun N-terminal kinase (JNK), and p38. After activation of the MAPKs by UV, transcription factor (AP-1) expression increases, leading to collagen fragmentation, an inflammatory response, and cell death [9,10]. In addition, NF-κB is another critical transcription factor of MMP in the dermis. In photodamaged conditions, NF-κB is translocated into the nucleus by degradation of IκB and elevates MMP and pro-inflammatory cytokines such as TNF-α, IL-1β, and IL-6 [11,12]. Interestingly, UVB alters not only the collagen degradation pathway but also the synthesis pathway, TGFβ/Smad. Concerning the TGFβ/Smad pathway, previous studies have reported that the TβRII level is reduced, and phosphorylation of Smad3 in the downstream of signaling is significantly decreased in photoaged human skin [13,14]. Impairment of the TGFβ/Smad pathway is also one of the mechanisms causing loss of collagen. Consequently, due to complicated changes in various pathways, the collapse of ECM occurs, resulting in wrinkle formation.

As described above, many studies on skin damage by UVB have been reported continuously, and investigations have examined anti-photoaging. In the cosmetics market, existing cosmetic products contain inorganic and chemical components. In response to this, studies regarding the safety and skin irritation of chemical components have been reported [15,16]. Therefore, much research is being conducted to find anti-photoaging effects from existing natural products and organic compounds. *Acer tataricum* subsp. *ginnala* (AG) is a herbal plant distributed in Korea, China, Japan, Mongolia, and East Russia. Regarding the effect of AG, several previous studies have been conducted. AG suppresses nitric oxide (NO) production and 2,2-diphenyl-1-picrylhydrazyl (DPPH) radical scavenging in lipopolysaccharide (LPS)-stimulated RAW264.7 cells [17]. Wu et al. reported that an active compound of AG exhibits a colorectal cancer chemoprevention effect via upregulating the nuclear factor E2-related factor 2(Nrf2)/heme oxygenase-1 (HO-1) signaling pathway that also contributes to inhibiting oxidative damage [18]. Regarding the anti-oxidative and anti-inflammatory activities, in an atopic dermatitis-induced mouse model, serum IgE and inflammatory cytokines, which affect skin lesions, were reduced by AG [19]. As above, it is reported that AG has anti-inflammatory, anti-cancer, and intense anti-oxidative activity. In addition, AG has long been used as a material for tea in China. For this reason, it is also called Ku-jin tea as other names in Chinese. It is known as a natural product that is caffeine-free and has high polyphenols and noticeable antioxidant activities. This plant has been used in traditional medicine for wound healing, arthritis, eye diseases, and diarrhea [20]. Even though AG is known for various efficacies, AG’s anti-photoaging effect is not yet well defined. Therefore, we investigate AG’s anti-photoaging effect and molecular mechanism in UVB-irradiated human dermal fibroblasts.

## 2. Results

### 2.1. Ultra-Performance Liquid Chromatography (UPLC) Quantitative Analysis

**Figure 1:**
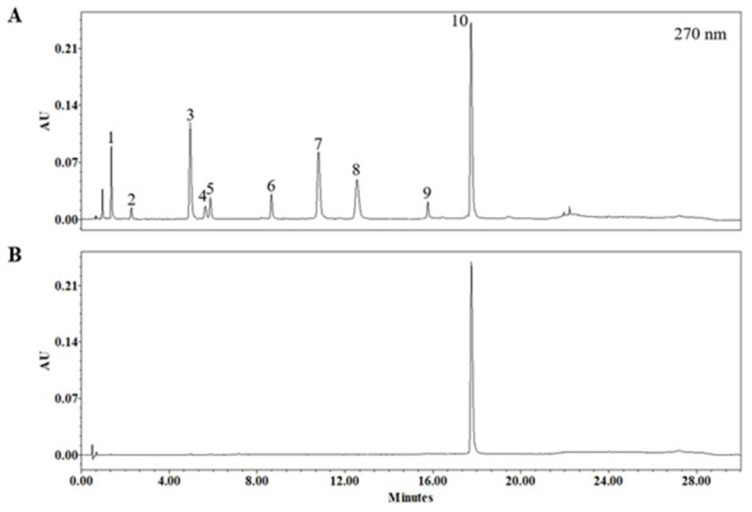
Ultra-performance liquid chromatography (UPLC) chromatogram of Acer tataricum subsp. ginnala extract (**A**) and reference standards solution ginnalin A (**B**). Among the 10 identified peaks, peak no. 10 was identified as ginnalin A. Detailed information of the other peaks are listed in Table 1.

The chromatogram of *Acer tataricum* subsp. *ginnala* detected at 270 nm is shown in Figure 1A. Figure 1B is a chromatogram of the reference standard Ginnalin A at 270 nm. The 10th peak was confirmed by direct comparison with the reference standard of Ginnalin A. Other peaks were identified by retention time, mass spectra, and UV-vis spectra with data from a previous study [20]. Detailed data for these are listed in Table 1. The calculated content of Ginnalin A in the extract was 12.374 ± 0.211% (Table 2).

### 2.2. Cell viability after AGE Treatment in HDFs

**Figure 2:**
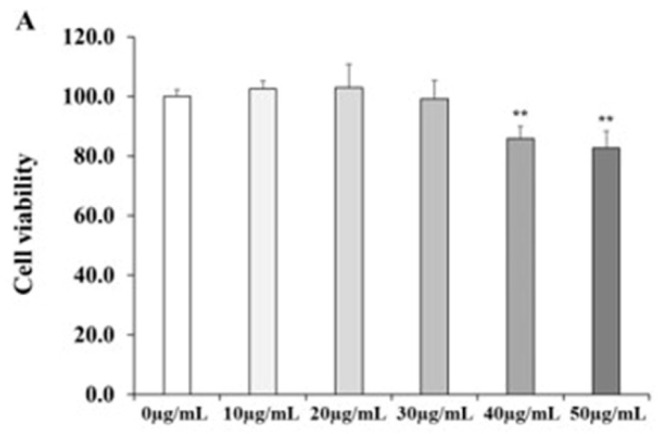
Cell viability after AGE treatment in HDFs. HDFs were treated with different concentrations of AGE, and incubated for 24 h. After incubation, MTT assay was performed to measure the cell viability of AGE (**A**). All data were expressed as the mean ± SD of at least three independent experiments. ** *p* < 0.01 versus control group.

We conducted an MTT assay to examine the cytotoxicity after AGE treatment (Figure 2). AGE was treated with diverse concentrations (10, 20, 30, 40, and 50 µg/mL) to HDFs. We determined that there was no significant change in the cell viability of HDFs up to 30 µg/mL of AGE. The group treated with 40 µg/mL of AGE decreased to 86% of the control group. Therefore, we set the concentrations (7.5, 15, and 30 µg/mL) of AGE in this research.

### 2.3. Effect of AGE on the Expression of Type I Procollagen and MMP1 in UVB-Irradiated HDFs

**Figure 3:**
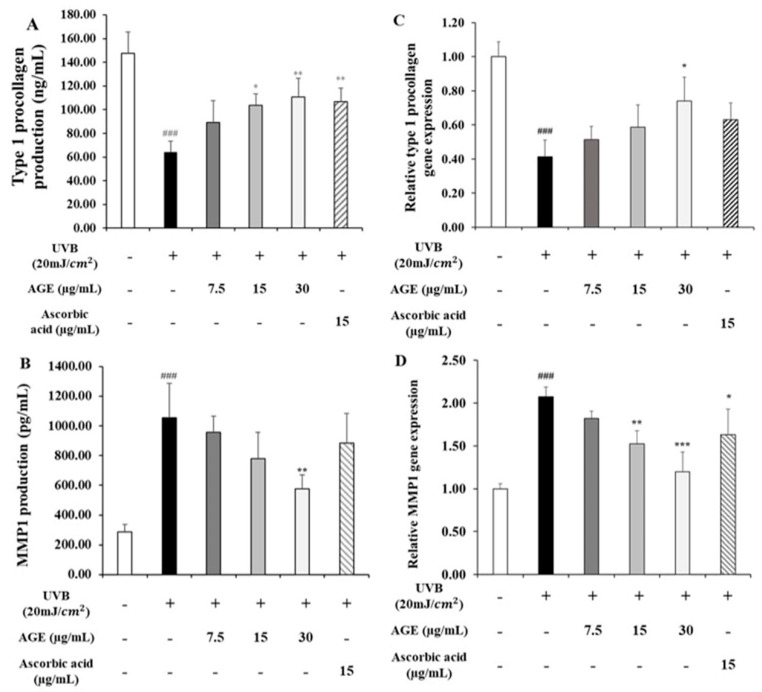
Effect of AGE on type I procollagen and MMP-1 level in UVB-irradiated HDFs. HDFs were irradiated with UVB (20 mJ/cm^2^), followed by treatment with 7.5, 15, and 30 µg/mL of AGE. After 24 h of incubation, detection of type I procollagen (**A**) and MMP-1 (**B**) production were performed by using ELISA kits. Gene expression of COL1A1 (**C**) and MMP-1 (**D**) were determined by quantitative real-time (qRT)-PCR. All data were expressed as the mean ± SD of at least three independent experiments. ### *p* < 0.001 versus untreated cells, * *p* < 0.05, ** *p* < 0.01, and *** *p* < 0.001 versus UVB-irradiated cells.

We measured the type I procollagen and MMP1 level that are the main factors related to skin aging in HDFs (Figure 3). The amount of type I procollagen in UVB-irradiated HDFs was reduced to 0.4-fold of the untreated group. After AGE treatment to photodamaged HDFs, the type I procollagen level was recovered dose-dependently. By contrast, the MMP1 level was increased by 3.6-fold of the untreated group. AGE also downregulated the level of MMP1 dose-dependently. The gene expression results showed similar patterns as above. The mRNA expression of type I procollagen was decreased by 0.4-fold of the untreated controls in the UVB-irradiated group. In AGE-treated groups, the gene expression of type I procollagen increased dose-dependently by 54%. In addition, in UVB-exposed HDFs, MMP1 gene expression was upregulated by 2.1-fold of the untreated group. AGE decreased the expression of MMP1 by 80%. Therefore, the results suggest that AGE has a protective effect against photoaging in UVB-irradiated HDFs.

### 2.4. Effect of AGE on MAPKs Expression in UVB-Irradiated HDFs

**Figure 4:**
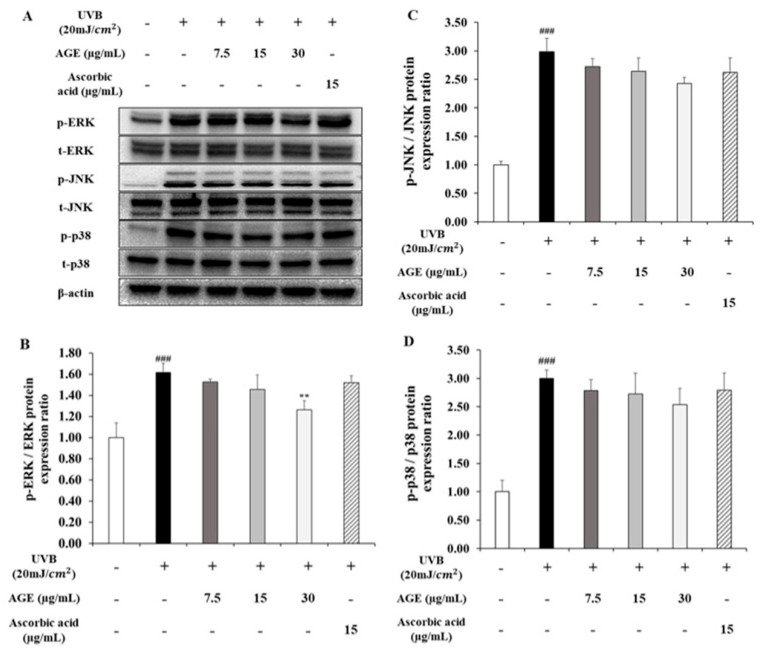
Effects of AGE on MAPK signaling pathway in UVB-irradiated HDFs. HDFs were irradiated with UVB (20 mJ/cm^2^), followed by treatment with 7.5, 15, and 30 µg/mL of AGE. After 24 h incubation, protein extraction was performed. Phosphorylation and total protein ratios of ERK (**B**), JNK (**C**), and p38 (**D**) were measured using Western blot (**A**). All data were expressed as the mean ± SD of at least three independent experiments. ### *p* < 0.001 versus untreated cells, ** *p* < 0.01 versus UVB-irradiated cells.

We determined the change of phosphorylated forms of the MAPKs such as ERK, JNK, and p38 to figure out the further effect of AGE on UVB-induced photoaging (Figure 4). In UVB-irradiated HDFs, phosphorylation of ERK, JNK, and p38 was stimulated by 1.6, 3.0, and 3.0-fold, respectively, compared to nonirradiated HDFs. These results suggest that HDFs are damaged by UVB through activating the MAPK signaling pathway. AGE significantly reduced ERK phosphorylation by 60% at 30 µg/mL compared with the UVB-induced group. In addition, the expression of phosphorylated JNK and p38 diminished in the group treated with 30 µg/mL AGE by 28% and 23%, respectively, but not significantly. As a result, we found that ERK phosphorylation was decreased by AGE significantly. AGE also downregulated the phosphorylated form of JNK and p38; however, there was no statistically significant difference.

### 2.5. Effect of AGE on AP-1 Expression in UVB-Irradiated HDFs

**Figure 5:**
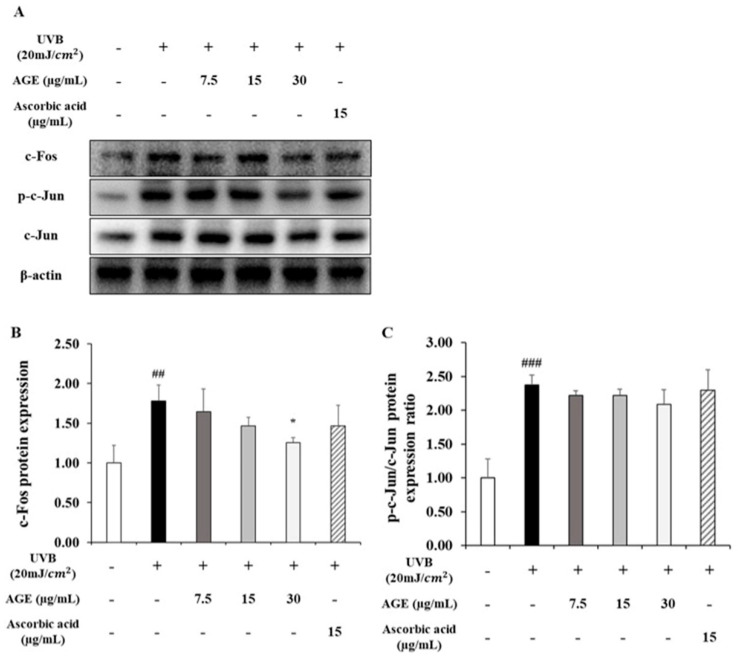
Effects of AGE on the expression of AP-1 in UVB-irradiated HDFs. HDFs were irradiated with UVB (20 mJ/cm^2^), followed by treatment with 7.5, 15, and 30 µg/mL of AGE. After 24 h of incubation, protein extraction was performed. Protein expressions of c-fos (**B**), c-jun, and p-c-jun (**C**) were determined by Western blot (**A**). All data were expressed as the mean ± SD of at least three independent experiments. ## *p* < 0.01 and ### *p* < 0.001 versus untreated cells, * *p* < 0.05 versus UVB-irradiated cells.

As shown in Figure 5, in the UVB-exposed group, c-Fos expression, which is the subunit of AP-1, was stimulated by 1.8-fold compared to the untreated group. By contrast, c-Fos expression was decreased by 67% in 30 µg/mL of AGE. c-Jun, another subunit of AP-1, is activated by 2.4-fold of the nonirradiated group. However, c-Jun phosphorylation was decreased by 22% in the 30 µg/mL of AGE. Therefore, it can be concluded that AGE decreased c-Fos expression significantly. AGE also downregulated the phosphorylated form of c-Jun; however, there was no statistically significant difference.

### 2.6. Effect of AGE on NFκB Pathway in UVB-Irradiated HDFs

**Figure 6:**
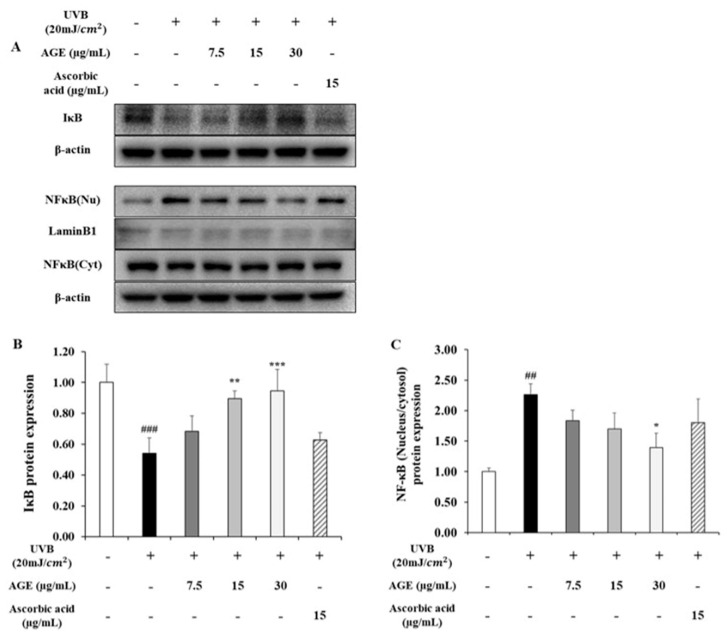
Effects of AGE on the expression of NF-κB signaling pathway in UVB-irradiated HDFs. HDFs were irradiated with UVB (20 mJ/cm^2^), followed by treatment with 7.5, 15, and 30 µg/mL of AGE. After 24 h of incubation, protein extraction was performed. Protein expressions of IκB (**B**), nucleus, and cytosolic NF-κB (**C**) were determined by Western blot (**A**). All data were expressed as the mean ± SD of at least three independent experiments. ## *p* < 0.01 and ### *p* < 0.001 versus untreated cells, * *p* < 0.05, ** *p* < 0.01, and *** *p* < 0.001 versus UVB-irradiated cells.

**Figure 7:**
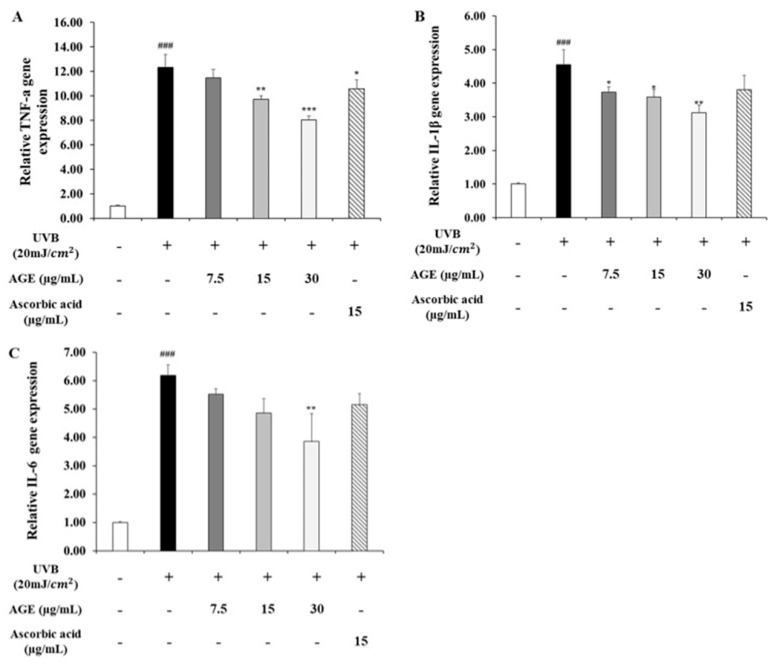
Effects of AGE on the expression of inflammatory cytokines in UVB-irradiated HDFs. HDFs were irradiated with UVB (20 mJ/cm^2^), followed by treatment with 7.5, 15, and 30 µg/mL of AGE. After 24 h of incubation, gene expressions of TNFα (**A**), IL1β (**B**), and IL6 (**C**) were measured by qRT-PCR. All data were expressed as the mean ± SD of at least three independent experiments. ### *p* < 0.001 versus untreated cells, * *p* < 0.05, ** *p* < 0.01, and *** *p* < 0.001 versus UVB-irradiated cells.

To determine the inhibitory effect of AGE in skin photoaging, we determined the NFκB level, which upregulates the expression of MMP1 and pro-inflammatory cytokines (Figure 6). In the UVB-irradiated group, IκB protein expression decreased by 0.5-fold of the untreated group; however, expression of IκB in the AGE-treated group was recovered significantly. For this reason, nucleus translocation of NF-κB was increased 2.3 times in the UVB-damaged state. However, the ratio of the NF-κB expressions in the nucleus and cytosol was reduced by 34, 44, and 69% in 7.5, 15, and 30 µg/mL of AGE, respectively. In addition, AGE restores the expression of IκB significantly more than ascorbic acid. Moreover, we investigated the change in gene expression of pro-inflammatory cytokines, TNF-α, IL-1β, and IL-6 (Figure 7). In the UVB-exposed group, mRNA expression of TNF-α, IL-1β, and IL-6 increased by 11.5, 4.6, and 6.2-fold, respectively. In comparison with the UVB-irradiated group, 30 µg/mL of the AGE showed reduction in the level of TNF-α, IL-1β, and IL-6 by 39%, 33%, and 40%, respectively. These data suggest that AGE downregulates the MMP1 and pro-inflammatory cytokines level via the NF-κB pathway.

### 2.7. Effect of AGE on TGFβ/Smad Pathway in UVB-Irradiated HDFs

**Figure 8:**
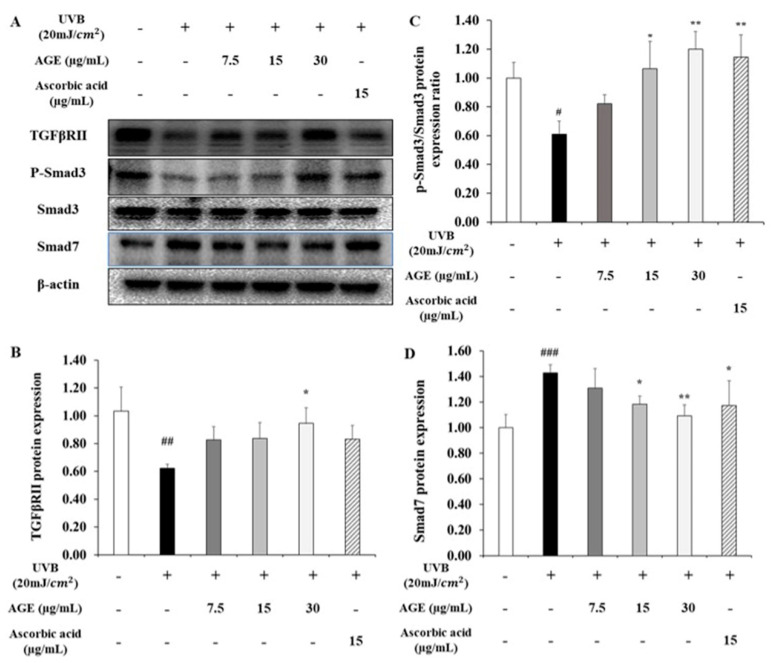
Effects of AGE on the expression of TGFβ/Smad signaling pathway in UVB-irradiated HDFs. HDFs were irradiated with UVB (20 mJ/cm^2^), followed by treatment with 7.5, 15, and 30 µg/mL of AGE. After 24 h of incubation, protein extraction was performed. Protein expressions of TGFβ receptor II (**B**), p-Smad-3, Smad-3 (**C**), and Smad-7 (**D**) were determined by Western blot (**A**). All data were expressed as the mean ± SD of at least three independent experiments. # *p* < 0.05, ## *p* < 0.01, and ### *p* < 0.001 versus untreated cells, * *p* < 0.05 and ** *p* < 0.01 versus UVB-irradiated cells.

When the skin is exposed to UV, collagen degradation is caused not only by the upregulated MAPK/AP-1 pathway but also by the impaired TGFβ/Smad pathway. Therefore, we confirmed the alteration of the TGFβ/Smad pathway after AGE treatment in UVB-irradiated HDFs (Figure 8). The TβRII protein expression diminished by 0.6-fold of the untreated group due to UVB irradiation. As we treated 7.5, 15, and 30 µg/mL of AGE in UVB-induced HDFs, TβRII expression was recovered by 49, 51, and 78%, respectively. In addition, phosphorylation of Smad3 was significantly increased by AGE treatment compared to the UVB-irradiated group. In particular, 15 and 30 µg/mL of AGE, and 15 µg/mL of ascorbic acid normalized the phosphorylation ratio of Smad3. Additionally, AGE diminished Smad7 expression, inhibitory Smad. In 30 µg/mL of the AGE-treated group, Smad7 expression was decreased markedly by 79%. These results demonstrated that AGE is effective in anti-photoaging by regulating the TGFβ/Smad pathway.

## 3. Discussion

Skin, the most voluminous organ in the human body, is susceptible to outer environmental components. This aspect makes the skin more vulnerable to hazardous factors and leads to appearing hallmarks of skin aging [21,22]. Particularly, UVB reaches the skin directly and increases the ROS level with small amounts of irradiation, so it is hazardous biologically to human skin [1]. In the present study, we identify the major compound of AG, Ginnalin A. It is a bioactive compound with diverse physiological functions, anti-oxidant, anti-cancer, and anti-inflammatory effects [17,23,24]. In human keratinocytes, Ginnalin A exerts ROS scavenging activity against oxidative stress [23]. Moreover, it has been reported that AG mainly contains polyphenol and gallotannin-based substances such as gallic acid, maplexin, and 3,6-di-O-galloyl-1,5-anhydro-D-glucitol [24]. Regarding skin aging, gallic acid has been reported to inhibit collagen degradation caused by collagenase. In addition, it promotes MMP1 reduction and upregulation of TGFβ1 and type I procollagen in UVB-irradiated HDFs and hairless mice [25]. Other gallotannins (Maplexin and 3,6-di-O-galloyl-1,5-anhydro-D-glucitol) have been reported to be anti-inflammatory and photo-protective [17,26]. Therefore, we predicted that various compounds from AG exert photo-protective effects in UVB-irradiated HDFs based on previous research.

The mechanical resilience of skin is significantly influenced by ECM. It is mainly composed of collagen fiber, especially type I, the most abundant structural protein. As the skin is exposed to UVB, MMP1, a major collagenase for type I procollagen, is upregulated, leading to collagen decomposition and wrinkle formation [22]. In this study, we found that AGE inhibits skin photoaging through recovering type I procollagen and decreasing MMP1. Then, to demonstrate the effect of AGE, we investigated the alteration of photoaging mechanisms by dividing into collagen degradation and collagen synthesis.

UVB irradiation changes amino acid residues into proteins. It can alter protein structure and function, leading to the modification of signaling cascades [27,28]. Typically, ROS production by UVB changes receptor tyrosine kinase (RTK), the cell surface receptor for growth factors and pro-inflammatory cytokines such as TNF-α, IL-1, and IL-6 [29]. As usual, RTK-related signaling cascades are preserved with the basal state by receptor protein tyrosine phosphatase (RPTP). However, it has been reported that ROS, occurring by UVB, reacts with the catalytic site of RPTP and leads to the inactivation of RPTP, thereby stimulating RTK-related signaling pathways [21]. Among them, the MAPK/AP-1 pathway is primarily relevant to the skin photoaging process. MAPKs are constituted with ERK, JNK, and p38. Phosphorylated ERK plays a critical role in the trigger for c-Fos induction [30,31]. Phosphorylated JNK and p38 accelerate c-Jun phosphorylation and promote its transactivation activity; then, c-Jun combines to target gene promoters as heterodimers, AP-1, with c-Fos [32,33]. Finally, AP-1 promotes MMP1 expression and collagen degradation [34,35,36]. In this study, we confirmed that AGE showed inhibitory effects on the MAPK/AP-1 pathway in UVB-irradiated HDFs. AGE downregulated ERK, JNK, and p38 phosphorylation overall. Then, the reduction of MAPK phosphorylation by AGE decreased AP-1 activity. In our study, AGE notably decreased ERK phosphorylation and c-Fos expression by 60 and 67%, respectively. In addition, JNK, p38, and c-Jun were similarly decreased by 28, 23, and 21%, respectively, in AGE-treated HDFs. We focused on the difference in recovery rates approximately two times between c-Fos and c-Jun after AGE treatment. Thus, it can be concluded that AGE inhibited UVB-induced MAPKs phosphorylation and AP-1 activity in UVB-damaged HDFs. Moreover, AGE suppressed ERK and c-Fos activity, especially. Regarding the inhibition of the ERK pathway, it has been reported that gallotannin reduced MMP1 expression by inhibiting ERK activity in human keratinocytes [37]. In AGE, various gallotannins were identified, so these multi-components would be involved in inhibiting photoaging. Ginnalin A, one of the gallotannins, was detected as a major compound of AGE, and we predicted it as an active compound. However, it is unclear which component mainly contributes to the inhibition of photoaging. Based on these results, further studies are needed to investigate the active compound to exert the anti-photoaging effect.

Meanwhile, NF-κB is generally known as a mediator of the inflammatory response and apoptosis induced by stimuli such as inflammatory cytokines, pathogens, oxidative stress, and UV irradiation. In terms of collagen degradation, it is another pivotal modulator for the transcriptional activity of MMP1 with MAPK/AP-1 in response to UVB irradiation [31]. NF-κB is controlled by combining with IκB, which inhibits NF-κB localization into the nucleus. However, UVB promotes IκB phosphorylation, and phosphorylated IκB is degraded by the proteasomal ubiquitination process [12]. Due to this alteration, NF-κB is translocated into the nucleus and upregulates MMP1 [38]. In our study, we figured out that AGE significantly restored IκB expression, which was damaged by UVB. IκB expression that was increased by AGE inhibits NF-κB translocation into the nucleus. These findings indicate that AGE can suppress the expression of MMP1 by modulating NF-κB signaling in addition to MAPK/AP-1 signaling in UVB-irradiated HDFs.

In this pathway, pro-inflammatory cytokines, TNF-α, IL-1β, and IL-6, are mainly involved in the photodamage with autocrine and paracrine reaction. In the UVB-irradiated group, levels of cytokines were increased significantly. According to the literature, in photoaged skin, TNF-α is responsible for the UVB-induced inflammatory response through the accelerating MAPK/AP-1 pathway and degrading IκB in the NF-κB pathway [39]. Regarding IL-1β in the photoaging, it has been reported that IL-1β impairs the TGFβ/Smad pathway by targeting TβRII and Smad7 [40]. Moreover, IL-6 also promotes the inflammatory response and causes thickened stratum corneum in the skin [41]. However, we confirmed that AGE reduced levels of TNF-α, IL-1β, and IL-6. It seems that AGE has the inhibitory effect on skin photoaging as it even controls the inflammatory response via regulating the NF-κB pathway. In addition, we noted a difference in the recovery of IκB and NF-κB comparing AGE and ascorbic acid. It is assumed that this gap leads to the distinct recovery rate of MMP1 and pro-inflammatory cytokines in AGE and ascorbic acid-treated groups.

In this study, AGE significantly restored the ERK and NF-κB pathway impaired by UVB. Kook et al. reported that MMP-1 is controlled by ERK-NF-κB signaling pathways in human periodontal ligament fibroblasts. Based on the present study and previous paper, we confirmed that AGE regulates collagen degradation via inhibiting ERK and NF-κB pathways [42].

Concerning collagen synthesis in the skin, the TGFβ/Smad pathway is a representative regulator to promote collagen production. Thus, dysregulation of this pathway can seriously impact collagen homeostasis. To initiate this process, TβRII dimerizes with TβRI. It causes TβRI phosphorylation, which in turn, recognizes and phosphorylates R-Smad, Smad3. The phosphorylated Smad3 is localized in the Smad Binding Element (SBE) of the nucleus with co-Smad, Smad4, thereby contributing to procollagen synthesis [43]. Based on this, we investigated whether AGE restores collagen synthesis-related factors damaged by UVB. After UVB irradiation, expression of TβRII was downregulated. However, AGE recovered the TβRII level in the photodamaged state. In the downstream, we found that AGE upregulated Smad3 phosphorylation in a dose-dependent manner. This means that the TGFβ/Smad signaling, which was impaired by UVB, was recovered by AGE. In addition, Smad7, an inhibitory Smad that blocks phosphorylation of Smad3 by TβRI, was upregulated by photodamage. Upregulation of Smad7 also causes the lack of TGFβ/Smad signaling transduction [14,44,45]. In this study, AGE downregulated Smad7 expression markedly, thus helping Smad3 phosphorylation. These results demonstrate that AGE can stimulate collagen synthesis by upregulating the TGFβ/Smad pathway and inhibiting Smad7 activation.

## 4. Materials and Methods

### 4.1. Preparation of Acer tataricum subsp. ginnala Extract (AGE)

*Acer tataricum* subsp. *ginnala* bark used in this study was collected in November 2019 at Gyeonggi-do in South Korea. It was cleaned, dried, and pulverized. Then, 100 g of *Acer tataricum* subsp. *ginnala* bark was extracted by boiling in 1 L of distilled water for 2 h with a reflux extractor. Then, AGE was filtered using Whatman filter paper (GE Healthcare, Buckinghamshire, UK) and evaporated under vacuum. After evaporation, the extract underwent the freeze-drying process and was stored at −20 °C. The yield of AGE was 15.2%. The voucher specimen of *A. tataricum* (KHUP-0327) was deposited at the Herbarium of College of Pharmacy, Kyung Hee University.

### 4.2. UPLC-PDA-ESI-MS Analysis

HPLC-grade formic acid was purchased from Wako (Osaka, Japan) and HPLC-grade acetonitrile was obtained from Fisher Scientific Korea (Seoul, Korea). HPLC-grade water was obtained from Duksan Pure Chemical Co. (Ansan, Gyenggi-do, Korea). Ginnalin A was purchased from Carbosynth Ltd. (Berkshire, UK) and the purity was >95%. A Waters AcquityTM H-class ultra-performance liquid chromatography (UPLC) system (Waters Corp., Milford, MA, USA) with a photodiode array (PDA) detector and JMS-T100TD (AccuTOF-TLC) (JEOL Ltd., Tokyo, Japan) spectrometer equipped with electrospray ionization (ESI) source was used for chromatographic and spectrometric (MS) analysis.

The chromatographic separation was carried out on an ACQUITY UPLC BEH C18 Column (130 Å, 1.7 µm, 2.1 mm × 50 mm, Waters Corp., Milford, MA, USA) attached with an ACQUITY UPLC BEH C18 VanGuard Pre-column (130Å, 1.7 µm, 2.1 mm × 5 mm). The mobile phase consisted of 0.1% formic acid in acetonitrile (solvent A) and 0.1% formic acid in water (solvent B). The gradient condition of the mobile phase was 0–2 min, 1%; 2–7 min, 1% to 6%; 7–14 min, 6%; 14–14.1 min, 6% to 11%; 14.1–20 min, 11%; 20–25 min, 11% to 100%; 25–27.5 min, 100% to 1%; 27.5–30 min, 1% as percent of solvent A. The flow rate was 0.3 mL/min and the column oven temperature was maintained at 30 °C. The injection volume was 1.5 μL. The representative chromatogram was extracted at 270 nm wavelength (Figure 1). The conditions of MS analysis in the negative ion mode were as follows: Scan range, m/z 100–2000; desolvating chamber temperature, 250 °C; orifice1 temperature, 80 °C; orifice 1 voltage, −80 V; orifice 2 voltage, −15 V; ring lens voltage, −15 V; peak voltage, 1000 V; detector voltage, 2000 V; nitrogen gas flow rate, 1.0 (nebulizing gas) and 3.0 L/min (desolvating gas). The extract powder of *Acer tataricum* subsp. *ginnala* was dissolved in distilled water and adjusted to a concentration of 2 mg/mL. The sample solution was filtered through a 0.2 μm polyvinylidenefluoride syringe filter (Whatman, Maldstone, UK) before being injected to the UPLC system. The chromatogram of *Acer tataricum* subsp. *ginnala* was detected at 270 nm.

### 4.3. UPLC Quantitative Analysis

A standard solution was prepared by dissolving Ginnalin A in 50% methanol to set a final concentration of 500 mg/L, and then filtering it through a 0.2 μm polyvinylidenefluoride syringe filter (Whatman, Maldstone, UK) and diluting at five concentrations of 31.25, 62.5, 125, 250, and 500 mg/L, which were used to calculate the regression equation for quantification. The coefficient values (r^2^) were 0.999. The precision was determined in a sextuplicate measurement of each standard. The relative standard deviations (RSD) were smaller than 0.59%. Using the established calibration curves, the contents of Ginnalin A in the extract were quantified.

### 4.4. Cell Culture

HDFs were purchased from American Type Culture Collection (ATCC; Manassas, VA, USA) and cultured with Dulbecco’s modified Eagle’s medium (DMEM, Welgene, Korea) containing 10% fetal bovine serum (FBS) (Hyclone, UT, USA) and 1% penicillin-streptomycin (Welgene, Korea). The cells were cultured in a 37 °C, 5% CO_2_ incubator. Culture media were changed every three days, and the cells were subcultured when the cells were grown to 8–90% confluence. All experiments were performed with HDFs between 5 and 10 passages.

### 4.5. UVB Irradiation and AGE Treatment

The UVB dose was based on the cell viability of HDFs, about 6–70% of the untreated group. Therefore, 20 mJ/cm^2^ was selected. Before UVB irradiation, HDFs were washed with phosphate-buffered saline (PBS) twice. The media were replaced with PBS when HDFs were exposed to UVB (20 mJ/cm^2^) (306 nm, G8T5E, 8W, Sankyo Denki, Japan) except the untreated group. Then, HDFs were incubated in serum-free media with or without different concentrations of AGE for 24 h. The positive control was 15 μg/mL of L-ascorbic acid (Sigma-Aldrich, St. Louis, MO, USA). All of the cell experiments were performed at least three times.

### 4.6. Cell Viability Assay

To measure the cell viability of AGE, HDFs were seeded at a density of 1 × 10^4^ cells on each well of 96-well plates and incubated at 37 °C, 5% CO_2_. After incubation, the cells were treated with different concentrations of AGE dissolved in DMEM without serum, and then incubated for 24 h. After incubation, the media were changed with MTT reagent (500 μL, 0.5 mg/mL concentration), and HDFs were incubated for 3 h. Then, the reagent was suctioned, and 1 mL of dimethyl sulfoxide (DMSO, Duksan, Korea) was added to each well to dissolve the formazan crystal. The cell viability was measured at 540 nm by an ELISA microplate reader (Bio-Tek Instruments Inc., Winooski, VT, USA).

### 4.7. Determination of Type I Procollagen and MMP-1 Production

For type I procollagen and MMP1 determination, HDFs were seeded 3 × 10^4^ cells per well on 24-well plates. After treating AGE with serum-free media, the cells were lysed with lysis buffer contained in the ELISA kit to measure type I procollagen and MMP-1 level. The measurement of type I procollagen and MMP-1 was performed according to the ELISA kit protocol (Procollagen Type I C-peptide (PIP) EIA kit; Takara Bio Inc, Otsu, Japan/Human MMP1 ELISA kit; Abcam, Cambridge, MA, USA).

### 4.8. Quantitative Real-Time Polymerase Chain Reaction (qRT-PCR) Analysis

The total RNA of HDFs was isolated according to the protocol of the Easy-RED TM total RNA extraction kit (iNtRON Biotechnology, Gyeonggi-do, Korea). After RNA extraction, cDNA was synthesized using a cDNA synthesis kit (Takara, Shiga, Japan). Then, the qRT-PCR analysis was performed with the SYBR Premix Ex Taq (Takara, Shiga, Japan) with an ABI StepOnePlusTM Real-Time PCR machine (Applied Biosystems, MA, USA). Table 3 lists the primer sequences used in qRT-PCR analysis. All results were normalized to GAPDH.

### 4.9. Western Blot Analysis

The protein of HDFs was extracted using RIPA II Cell Lysis Buffer(1X) (GenDEPOT, USA) containing protease inhibitor cocktails (Roche, Mannheim, Germany) and PhosSTOP EASYpack phosphatase inhibitor cocktail tablets (Roche, Mannheim, Germany). To obtain supernatants, cell lysates were centrifuged at 13,000 rpm for 15 min at 4 °C. Nuclear and cytosol fractionation was performed according to the manufacturer’s instructions of the Nuclear Extraction Kit (Abcam, Cambridge, MA, USA). Protein concentration was measured using a PierceTM BCA Protein Assay Kit (Thermo Fisher Scientific, Waltham, MA, USA), and each protein was separated in the SDS-PAGE gel. Then, proteins were transferred to a polyvinylidene fluoride (PVDF) membrane. After blocking the membrane with 5% skim milk in tris-buffered saline with Tween 20 (TBST) for 1 h, the membrane was incubated overnight with primary antibody diluted with 5% BSA in TBST at 4 °C. The membrane was incubated with horseradish peroxidase-conjugated secondary antibodies in blocking solution (Santa Cruz Biotechnology Inc., Santa Cruz, CA, USA) for 2 h. All bands were visualized by using ChemiDoc^TM^ XRS+System (Bio-Rad, Richmond, CA, USA). Band intensity was analyzed with ImageJ software. The primary antibodies are as follows: Type I procollagen, MMP-1, and β-actin (Santa Cruz Biotechnology Inc., Santa Cruz, CA, USA), Smad-7 (R&D systems Inc., Minneapolis, USA), ERK, phosphorylated-ERK (p-ERK), JNK, phosphorylated-JNK (p-JNK), p38, phosphorylated-p38 (p-p38), Smad-3, phosphorylated-Smad-3 (p-smad3), c-fos, c-jun, phosphorylated-c-jun (p-c-jun), TGFβ receptor II, IκB, and NF-κB p65 (Cell Signaling Technology, Danvers, USA).

### 4.10. Statistical Analysis

All results performed were with at least three independent experiments. Statistical differences were determined using one-way analysis of variance (ANOVA) followed by Tukey’s post hoc test. Statistical Packages for Social Science (SPSS) 25 software (SPSS Inc., Chicago, IL, USA) was used to conduct statistical analysis. The data were presented as the mean ± standard deviation (SD). Statistical significance was set at # < 0.05, ## < 0.01, and ### < 0.001 compared to the untreated cells; * *p* < 0.05, ** *p* < 0.01, and *** *p* < 0.001 compared to the UVB-irradiated cells.

## 5. Conclusions

In this study, we demonstrated that AGE plays a protective role in both collagen breakdown and synthesis in UVB-irradiated HDFs. AGE inhibits collagen degradation by downregulating MAPK/AP-1 and NFκB pathways activated by UVB irradiation, and it leads to the reduction of MMP1 and pro-inflammatory cytokines. In particular, AGE can also promote collagen synthesis by restoring the TGFβ/Smad pathway damaged by UVB irradiation. Taken together, the results of this study suggest that AGE can function as one of the anti-photoaging materials.

## Figures and Tables

**Table 1:** Retention time (Rt), UV λ max, chemical formula, theoretical mass, measured mass, mass difference, and identification of UPLC peaks.

Peak No.	Rt (min)	UV λ max (nm)	Formula	[M − H]^−^ Theoretical Mass (m/z)	[M − H]^−^ Measured Mass (m/z)	Mass Difference (mmu)	Identification
1	1.514	220/271	C_7_H_6_O_5_	169.013698	169.01369	−0.008	gallic acid
2	2.892	215/274	C_13_H_16_O_9_	315.071607	315.07041	−1.197	Ginnalin B/C or Maplexin A/B
3	5.543	220/274	C_13_H_16_O_9_	315.071607	315.07066	−0.947	Ginnalin B/C or Maplexin A/B
4	6.241	214/277	C_20_H_20_O_13_	467.082566	467.08154	−1.026	Maplexin C/D or 3,6-di-O-galloyl-1,5-anhydro-D-glucitol
5	6.482	215/274	C_13_H_16_O_9_	315.071607	315.07072	−0.887	Ginnalin B/C or Maplexin A/B
6	9.200	215/277	C_20_H_20_O_13_	467.082566	467.08605	3.484	Maplexin C/D or 3,6-di-O-galloyl-1,5-anhydro-D-glucitol
7	11.433	220/274	C_20_H_20_O_13_	467.082566	467.08684	4.274	Maplexin C/D or 3,6-di-O-galloyl-1,5-anhydro-D-glucitol
8	13.270	217/275	C_20_H_20_O_13_	467.082566	467.08691	4.344	Maplexin C/D or 3,6-di-O-galloyl-1,5-anhydro-D-glucitol
9	15.970	217/275	C_20_H_20_O_13_	467.082566	467.08785	5.284	Maplexin C/D or 3,6-di-O-galloyl-1,5-anhydro-D-glucitol
10	17.764	224/274	C_20_H_20_O_13_	467.082566	467.08779	5.224	Ginnalin A (aceritannin)

**Table 2:** The regression equation, limit of detection (LOD), limit of quantification (LOQ), and quantification result of ginnalin A. In the regression equation, y represents the area of the ginnalin peak in the chromatogram monitored at 270 nm of the UV/Vis detector, and the x-axis unit is mg/L.

Compound	Regression Equation	R^2^	Linear Range (mg/L)	LOD (mg/L)	LOQ (mg/L)	Contents of Ginnalin A (%)
Ginnalin A	y = 6448.4x − 25435	0.9999	31.25–500	7.408	22.449	12.374 ± 0.211

**Table 3:** The oligonucleotide primer sequences used in RT-qPCR.

Gene	Sequences	
*COL1A1*	F: AGGGCCACGAAGACATC	R: AGATGACGTCATCGCACAACA
*MMP1*	F: CCCAAAAGCGTGTGACAGTAAG	R: CTTCCGGGTAGAAGGGATTTG
*IL-6*	F: GTGTGAAAGCAGCAAAGAGGC	R: CTTCCGGGTAGAAGGGATTTG
*IL1-β*	F: AAAAGCTTGGTGATGTCTGG	R: TTTCAACACGCAGGACAGG
*TNF-α*	F: CAAAGTAGACCTGCCCAGAC	R: GACCTCTCTCTAATCAGCCC
*GAPDH*	F: ACCCACTCCTCCACCTTTGA	R: TGGTGGTCCAGGGGTCTTAC

## Data Availability

The data presented in this study are available on request from the corresponding author.
